# Stable Deuterium Labeling of Histidine-Rich Lysine-Based Dendrimers

**DOI:** 10.3390/molecules24132481

**Published:** 2019-07-06

**Authors:** Nadezhda N. Sheveleva, Denis A. Markelov, Mikhail A. Vovk, Irina I. Tarasenko, Mariya E. Mikhailova, Maxim Yu Ilyash, Igor M. Neelov, Erkki Lahderanta

**Affiliations:** 1 Saint Petersburg State University, 7/9 Universitetskaya nab, 199034 Saint Petersburg, Russia; 2 Institute of Macromolecular Compounds, Russian Academy of Sciences, Bolshoi Prospect 31, V.O., 199004 Saint Petersburg, Russia; 3 Saint Petersburg National Research University of Information Technologies, Mechanics and Optics (ITMO University), Kronverkskiy pr. 49, 197101 Saint Petersburg, Russia; 4 Department of Physics, LUT University, Box 20, 53851 Lappeenranta, Finland

**Keywords:** peptide dendrimer, deuterium labeling, histidine

## Abstract

Peptide dendrimers, due to their biocompatibility and low toxicity, are highly promising candidates as nanocarriers for drugs and genes. The development of this kind of delivery system requires reliable monitoring of their metabolic and biological pathways. In this respect, hydrogen isotope labeling has tremendous importance, being a safe tool for detection of the labeled nanocarriers. In this work, we have synthesized new histidine-rich lysine-based dendrimers (Lys-2His dendrimer) with two linear histidine (His) residues in every inner segment. The presence of His residues has enabled us to perform controlled deuteration of Lys-2His dendrimers. The high deuteration degree (around 70%) does not practically change after redissolving the samples in H_2_O and heating them at 40 °C, which indicates the isotopic label stability.

## 1. Introduction

Dendrimers are hyperbranched, monodisperse macromolecules with a well-defined structure, multivalency, and nanoscale sizes. Dendrimers have great potential in biomedical applications due to their unique properties [1,2,3]. It has been shown that some types of dendrimers exhibit antibacterial, antiviral, and antitumor activity [4,5,6], but more often they are considered as nanocarriers for drugs and genes [2,7,8]. There is a huge variety of dendrimer structures that differ in core type, branching units, and surface functional groups. Many of the dendrimers have been investigated extensively and are already used in drug and gene delivery [1,9,10,11]. Poly-l-lysine (PLL) dendrimers have attracted considerable attention by many researchers who attempt to synthesize a safe and reliable dendrimer-based delivery system [12,13]. PLL dendrimers differ from synthetic analogs in their synthesis that is conducted using natural compounds (amino acid residues). Good biocompatibility and low toxicity are among the main favorable characteristics of these dendrimers [2,9,14]. NMR studies of PLL dendrimers were performed by us earlier [15,16,17,18]. In order to improve physicochemical and especially biological properties, the dendrimers are modified using various strategies, for example, surface functionalization, PEGylation, and acetylation [19,20]. One highly promising strategy for modifying bioactive molecules is the introduction the stable isotopes into their internal structure. The isotopes serve as a label for a compound that can be detected using NMR and mass spectrometry [21,22]. Among the most commonly used stable isotopes, deuterium finds important applications in the pharmaceutical industry for drug discovery and development [23,24].

Incorporation of His residues containing imidazole groups gives several advantages to a PLL dendrimer as a system for drug and gene delivery. The imidazole ring group has a p*K*_a_ of ~6.0 [25]. Since the imidazole group can be neutral or cationic at different pH values, histidine-rich molecules show the proton sponge effect that helps to overcome physiological barriers inside the cell [10,13,26,27,28]. Protonation of these groups leads to disruption of the endosomal membrane and early release of entrapped molecules [29]. Therefore, the high proton buffering capacity of histidine-modified dendrimers results in increasing their transfection efficiency which improves intracellular DNA transport [27].

One unique property of histidine residue is the possibility of its selective deuteration. C_2_ protons of the histidine imidazole ring are substituted by deuterium in heavy water (D_2_O) [30,31,32]. After removing D_2_O and redissolving peptide samples in water, labile deuterons in amine, imine, and amide NH groups were replaced by protons rapidly, while deuteration at the C_2_ atom of the imidazole ring was preserved [33]. In comparison with NH or NH_2_ groups, the isotope exchange reaction at the C_2_ atom of an imidazole ring occurs more slowly and has a half-life on the order of days [31,34]. Mobility of histidine residues and their accessibility to the aqueous solvent influence the rate of isotope exchange. The latter was higher in proteins where His residues were situated on the surface of the molecule [30,34,35]. Deuteration of l-histidine was carried out to synthesize deuterium-labeled peptides and proteins [32]. Moreover, peptides and proteins were deuterated to study their conformation, dynamics, and biological and metabolic pathways [31,33,36,37]. Proteins are completely denatured when exposed to high temperatures. Therefore, the range of temperatures used for their deuteration is severely limited. Generally, the hydrogen–deuterium exchange reaction in peptides and proteins containing histidine residues was conducted by incubating samples at 37–40 °C over 2–7 days [30,35,37,38].

The hydrogen–deuterium exchange reaction can be an invaluable tool for the preparation of deuterium-labeled peptide dendrimers. It should be noted that the synthesis of peptide dendrimers is a complex and multistage process. Intermediate products are purified after completion of each step of the synthesis. Significant reduction in deuterated histidine residues can occur under rather harsh synthesis conditions. At the same time, the overall yield of dendrimer after synthesis is about 30% [5]. Therefore, the use of pre-deuterated histidine residues can be resource-consuming.

**Figure 1:**
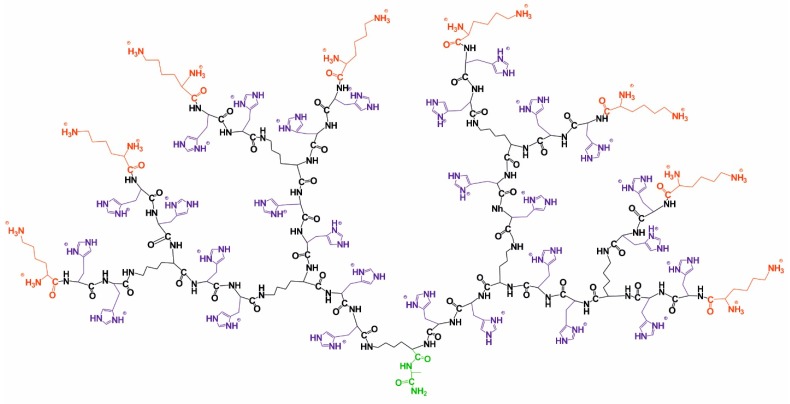
Structural formula of Lis-2His dendrimer. Green color marks the core, black corresponds to the main chain, violet marks the side segments, and red corresponds to the terminal segments.

Here, we have synthesized and deuterated a second generation lysine-based dendrimer by inserting two histidine amino acid residues into every inner segment (Figure 1). By analogy with our previous works [39,40], we have modified a dendrimer interior. The insertion of arginine or histidine amino acid residues into inner segments of lysine dendrimer could enhance its potential for drug and gene delivery (see, for instance, a recent review [19]). It is believed that the mechanism of this improved delivery is different. In particular, arginine residues allow more tight DNA packing. At the same time, histidine residues sensitize the delivery vehicles for acidic pH and manipulate the pH-based lysosomal escape. This work is devoted to the possibility of stable selective deuteration of Lys-2His dendrimers. The hydrogen–deuterium exchange at the C_2_ atoms of the histidine imidazole groups was used for controlled isotope labeling. We prepared deuterium-labeled histidine-containing lysine-based dendrimers using heavy water and by heating the samples. We showed that the labeling is stable at physiological conditions. These findings open new perspectives of biomedical applications of His-modified peptide dendrimers, especially in drug and gene delivery.

The paper is organized as follows. In Section 2, we describe the synthesis of a lysine-based dendrimer with double histidine residues and the preparation of samples for the experiments. Section 3 is devoted to the confirmation of the Lys-2His dendrimer structure by various NMR methods. In Section 4, we present the description of the deuteration process and the analysis of the obtained results. In the last section, we shortly summarize the results and conclusions.

## 2. Synthesis and Sample Preparation

Histidine-modified lysine-based dendrimer of the second generation (Lys-2His dendrimer) is shown in Figure 1. The core of Lys-2His dendrimer consists of alanine amino acid residue. The inner segments contain ε- or α-part of Lys and two His residues. The side segment consists of β-part of His residue containing imidazole group. The terminal segments contain Lys residues.

Lys-2His dendrimer was synthesized by the solid peptide phase reaction (SPPS) that has been performed manually in polypropylene syringes with a porous membrane on polymer support using the BOC-strategy with DIC/HOBt as a condensing mixture. Trifluoroacetic acid was used for deblocking at the acylation stage. An alanine residue was introduced at the C-terminus of the dendrimer. *N*ε, *N*α-di-(tert-butylhydroxycarbonyl)lysine was introduced into the branching points and, subsequently, double the amounts of amino acid derivatives were added. 4-*N*, *N*-dimethylaminopyridine (DMAP) was added to the reaction mixture as a catalyst for complete conversion on the last stage of the dendrimer growth. At the final stage of the synthesis, the target dendrimer molecule was cleaved from the polymeric carrier with complete deprotection of the TFMSA/TFA system in the presence of scavengers. Purification of the crude dendrimer was performed by gel-filtration on a Sephadex G-50 column (Pharmacia Fine Chemicals, Inc., Uppsala, Sweden). The corresponding fraction was dialyzed. The purification degree (95%) of the product was analyzed by RP-HPLC. A detailed description of the synthesis is presented in the Supplementary Materials (SM).

For the deuteration procedure, Lys-2His dendrimer was dissolved in D_2_O at a concentration of 1.55 g/dl with or without 0.157 M NaCl (saline solution).

## 3. H and ^13^C Spectral Characterization

NMR measurements were carried out on a Bruker Avance III 500 (Bruker BioSpin AG, Fällanden, Switzerland). One-dimensional ^1^H (500 MHz) and ^13^C (126 MHz) NMR spectra were recorded. We also performed two-dimensional ^1^H-^1^H COSY, ^1^H-^13^C HSQC, and HMBC NMR experiments for structural characterization of Lys-2His dendrimer using standard pulse sequences.

**Figure 2:**
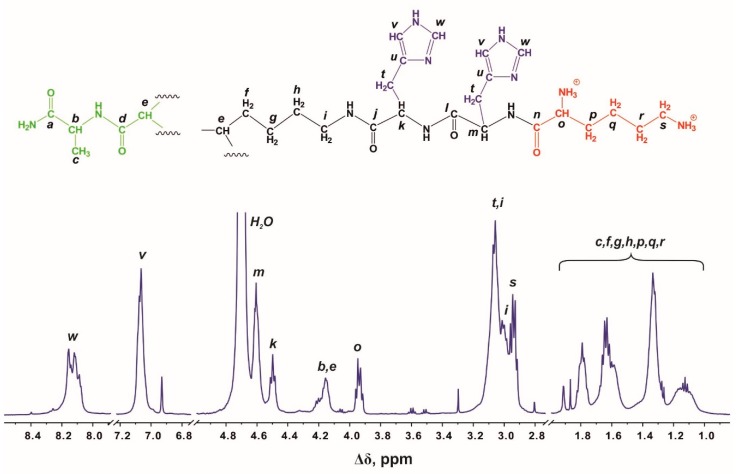
^1^H NMR spectrum of Lis-2His dendrimer in D_2_O at 25 °C.

There are four regions in the ^1^H NMR spectrum (Figure 2) where peaks are observed: the first region at 8.35–6.90 ppm refers to the protons of imidazole rings in His residues; the second region from 4.55 to 3.85 ppm refers to protons in CH groups; the third region from 3.25 to 2.82 ppm is attributed to protons in CH_2_ groups that are adjacent to nitrogen atoms; and the fourth region at 1.95–1.00 ppm refers to methyl and methylene groups. The ratios of the integrated areas of each type of protons match the expected values (Table 1).

**Figure 3:**
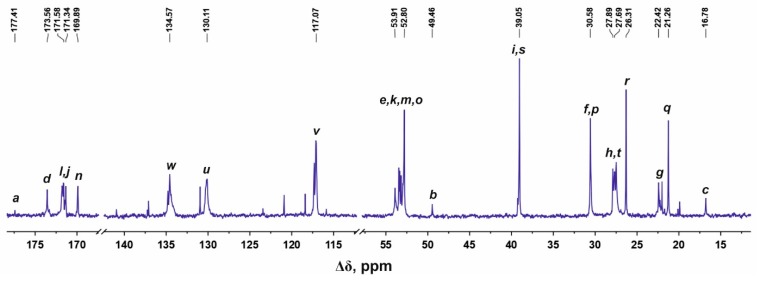
^13^C NMR spectrum of Lis-2His dendrimer in D_2_O at 25 °C. The letter symbols correspond to the designations of the groups in Figure 2.

Figure 3 shows the ^13^C NMR spectrum of the Lys-2His dendrimer. The signals in the range from 177.50 to 169.60 ppm refer to carbon atoms located in carboxyl groups. The peaks at 134.57, 130.11, and 117.07 ppm correspond to carbons in imidazole rings of His. The signals from carbons of CH groups are located in the region 54.20–49.00 ppm. The peak 30.95 ppm is attributed to the CH_2_ groups adjacent to nitrogen atoms in the inner and terminal Lys segments. The CH_2_ groups bonded to imidazole rings of His residues have the signal at about 27.50 ppm. In the region of 31.00–16.75 ppm there are peaks from carbons in the CH_2_ groups of the aliphatic part of the dendrimer.

We measured two-dimensional ^1^H-^1^H COSY, ^1^H-^13^C HSQC, and HMBC spectra for accurate correlation of peaks in the ^1^H and ^13^C NMR spectra. The detailed analysis of ^1^H-^13^C two-dimensional spectra and chemical shift assignments are provided in the SM. In conclusion of this section, we found that the claimed structure of Lis-2His dendrimer was totally confirmed by NMR spectroscopy (Figure 1).

## 4. Deuteration (Hydrogen–Deuterium Exchange)

Here, we present the results of deuteration of the histidine-modified lysine-based dendrimer. Samples of Lys-2His dendrimer were dissolved in heavy water and heated from 25 to 70 °C in increments of 5 °C. Samples were held at each temperature for 2 h. The deuterium incorporation was detected and confirmed by the proton NMR spectra which were recorded after each step.

**Figure 4:**
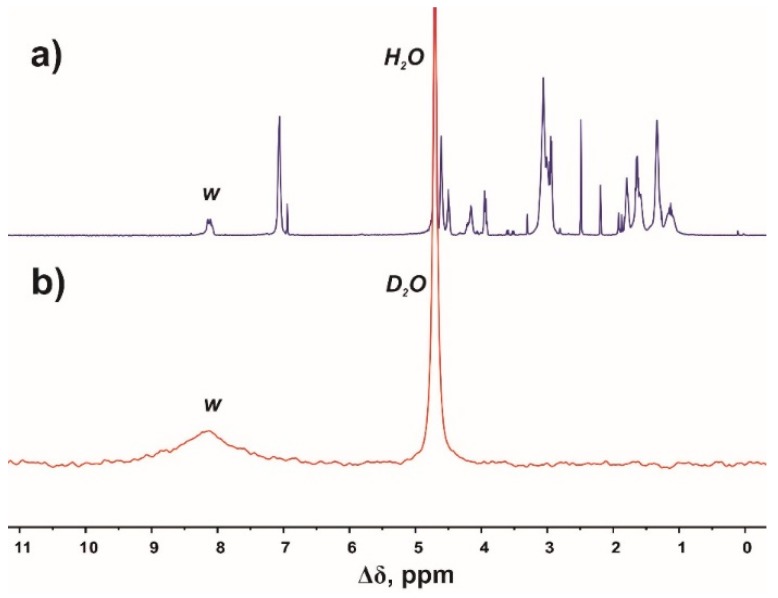
(**a**) ^1^H and (**b**) ^2^H NMR spectra of Lys-2His dendrimer in D_2_O after heating. ^2^H NMR spectrum was recorded using a pulse sequence that suppresses the solvent signal. The letter symbols correspond to the designations of the groups in Figure 2.

In Figure 4, we compare the ^1^H (Figure 4a) and ^2^H (Figure 4b) NMR spectra of Lys-2His dendrimer in D_2_O after heating. We are interested in the peak (*w*) with the chemical shift at 8.12 ppm that corresponds to the signal from protons at the C_2_ carbons of imidazole rings. As shown in Figure 4b, the signal from deuterons appears at 8.12 ppm. The presence of this peak in the ^2^H NMR spectrum indicates that substitution of hydrogens by deuterons at the C_2_ carbons of imidazole rings has occurred.

**Figure 5:**
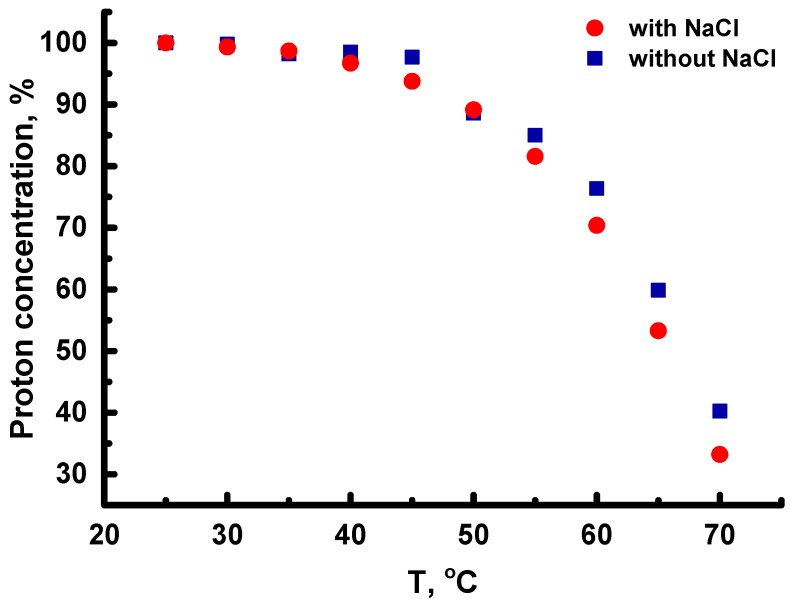
The temperature dependence of the concentration of protons at the C_2_ carbons of histidine imidazole rings (8.12 ppm) during heating.

The temperature dependence of the concentration of protons at the C_2_ carbons of imidazole rings in His residues is presented in Figure 5. As seen in Figure 5, the proton concentration decreases up to 30% after increasing temperature from 25 to 70 °C. Therefore, the deuteration degree at the C_2_ position in the imidazole rings is around 70%. This result indicates that His residues in the inner segments are available to the water solvent. This fact is in agreement with the data of the atomistic modeling for the dendrimer that we carried out earlier [41]. We found that in the absence of NaCl in the solvent, the deuteration process has practically the same dependence, but the maximal value of the deuteration degree is smaller (~60%) than in the saline solution. We think that this result is caused by the presence of the salt ions in the solvent. Particularly, chlorine ions were used in histidine deuteration experiments [32].

**Figure 6:**
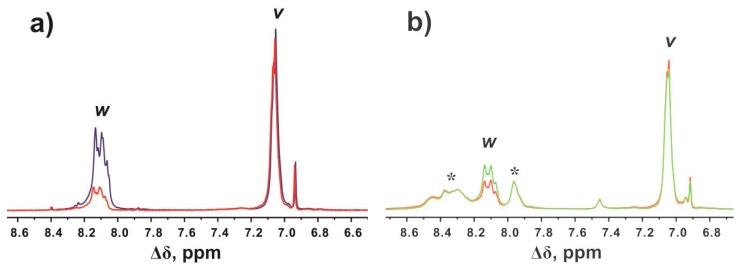
Evolution of the peak at 8.12 ppm from protons at the C_2_ carbons of the imidazole rings of histidine residues in Lys-2His dendrimer: (**a**) ^1^H NMR spectra of Lys-2His dendrimer in D_2_O before (blue) and after (red) heating; (**b**) ^1^H NMR spectra of Lys-2His dendrimer in H_2_O before (red) and after (green) heating. The peaks from protons in the NH groups are indicated by *.

It is important to determine that deuterium labels will be stable during biological and medical studies. We have to make sure that the deuterated C_2_ carbons of the imidazole rings in histidine residues do not undergo the back-exchange reaction under physiological conditions. For this purpose, we removed D_2_O from the solution and redissolved Lys-2His dendrimer in H_2_O. The concentration of NaCl in the solution was kept the same. Heating at 40 °C over four hours did not change the integral value of the peak (*w*) at 8.12 ppm. It means that the deuteration degree remains constant under physiological conditions. The replacement of deuterons with protons was observed when the dendrimer solution was heated at 70 °C over four hours. Figure 6 illustrates the evolution of the peak (*w*) at 8.12 ppm during hydrogen–deuterium exchange (a) and deuterium-hydrogen exchange (b) reactions. As can be seen in Figure 6b, recovery of the integral value of the peak (*w*) has partially occurred. It should be noted that the additional peaks at 7.95 and 8.30 ppm (Figure 6b) are assigned to protons in the NH groups and appeared as a result of the reverse exchange in these groups in H_2_O.

## 5. Conclusions

We synthesized a new histidine-modified lysine-based dendrimer using the solid peptide phase reaction. Lys-2His dendrimer was dialyzed, and its purification degree was analyzed by RP-HPLC. The claimed structure was confirmed and characterized by one- and two-dimensional ^1^H and ^13^C NMR spectroscopy. In particular, ^1^H-^1^H COSY, ^1^H-^13^C HSQC, and HMBC spectra were recorded and analyzed. It was established that isotope exchange occurs in Lys-2His dendrimer at the C_2_ carbons of the imidazole rings of histidine residues during heating. The hydrogen–deuterium exchange has been more intensive in the temperature range 55−70 °C in heavy water. The high level of deuteration (around 70%) was achieved. Deuterons were partially replaced by hydrogens at the C_2_ carbons of the imidazole ring of histidine residues after heating at 70 °C. However, the carbon at the C_2_ position in the imidazole rings remained deuterated in aqueous solution under physiological conditions, particularly up to 40 °C.

Here, we have demonstrated that it is possible to prepare a deuterium-labeled histidine-containing lysine-based dendrimer using a rapid and simple method without specific requirements. The chemical structure of Lys-2His dendrimer remains stable at relatively high temperatures during the isotope exchange reaction. We believe that the possibility of direct deuteration will expand the range of potential biological and medical applications of His-modified peptide dendrimers.

## Figures and Tables

**Table 1:** Chemical shift assignments and integral values.

Peak	Type of Group	Chemical Shift, ppm	Integral Value	Number of Protons in Groups
***w***	**CH**-(N) (in imidazole ring)	8.12	26	28
***v***	**CH**-(N) (in imidazole ring)	7.06	26	28
***m,k,b,e,o***	**CH**-(N)	4.64–3.88	44	44
***i,t,s***	**CH_2_**-(N)	3.22–2.83	86	86
***c,f,g,h,p,q,r***	**CH_2_**, **CH_3_***^a^*	1.93–1.00	102	90 + 3 *^a^*

^a^ In the core.

## Supplementary Materials

The following are available online. Figure S1: The principal scheme of the synthesis of Lys-2His dendrimer from the core to the first generation, Figure S2: ^1^H-^13^C HSQC spectrum of Lys-2His dendrimer in D_2_O at 25 °C. The letter symbols correspond to the designations of the groups in Figure 2, Figure S3: ^1^H-^13^C HMBC spectrum of Lys-2His dendrimer in D_2_O at 25 °C at the range from 5.0 ppm to 1.0 ppm. The letter symbols correspond to the designations of the groups in Figure 1, Figure S4: ^1^H-^13^C HMBC spectrum of Lys-2His dendrimer in D_2_O at 25 °C at the range from 8.5 ppm to 6.5 ppm. The letter symbols correspond to the designations of the groups in Figure 1.
