# Supplementary material for: Stable Deuterium Labeling of Histidine-Rich Lysine-Based Dendrimers
Source: Molecules. 2019 Jul 6;24(13):2481. doi: 10.3390/molecules24132481 (PMC6651089; doi:10.3390/molecules24132481)
Supplement: Supplementary file 1 [file molecules-24-02481-s001.pdf]

SUPPLEMENTARY MATERIALS

Stable Deuterium Labeling of Histidine-Rich Lysine-Based Dendrimers

Nadezhda N. Sheveleva[1], Denis A. Markelov[1*], Mikhail A. Vovk[1], Irina I. Tarasenko[2], Mariya E. Mikhailova[1], Maxim Yu. Ilyash[3], Igor M. Neelov[3], Erkki Lahderanta[4]

[1]*St. Petersburg State University, 7/9 Universitetskaya nab., St. Petersburg, 199034 Russia. Email: n.n.sheveleva@spbu.ru.*

[2]*Institute of Macromolecular Compounds, Russian Academy of Sciences, Bolshoi Prospect 31, V.O., St. Petersburg, 199004 Russia. Email: itarasenko@list.ru.*

[3]*St. Petersburg National Research University of Information Technologies, Mechanics and Optics (ITMO University), Kronverkskiy pr. 49, St. Petersburg, 197101 Russia. Email: i.neelov@mail.ru.*

[4]*Department of Physics, LUT University, Box 20, 53851 Lappeenranta, Finland. Email: erkki.lahderanta@lut.fi.*

[*]*Correspondence: markeloved@gmail.com*

Synthesis of Lys-2His dendrimer

Materials. Amino acids (L-lysine, L-histidine, L-alanine) were purchased from Iris Biotech GMBH (Germany). Trifluoromethanesulfonic acid (TFMSA), diisipropylcarbodiimide (DIC), 1-hydroxybenzotriazole (HOBt), thioanisole, ethanedithiol were purchased from Sigma-Aldrich (Germany) and used as received. Triethylamine (Et3N) and dichloromethane were purchased from Vecton Ltd. (Russia) and distilled before use. Dimethylformamide (DMF) was purchased from Vecton Ltd. (Russia), dried with 4 Å molecular sieves and distilled under vacuum. Trifluoroacetic acid (TFA) was purchased from Panreac (Spain) and distilled before application. All solvents were purified and distilled using standard procedures.

Instruments. Purification degree of the reaction product was assessed by RP-HPLC on Shimadzu LC-20 Prominence system (Japan) equipped with Luna C18 (2) column (5μm, 4.6 × 150 mm).

Synthesis and characterization. Lys-2His dendrimer were synthesized by the solid peptide phase reaction (SPPS) that were performed manually in polypropylene syringes with a porous membrane on a polymeric support, p-methylbenzhydrylamine resin (capacity 0.85 mmol/g) using the BOC-strategy, DIC/HOBt as a condensing mixture, and trifluoroacetic acid for deblocking at the acylation stage. To protect functional groups of amino acids, the tert-butylhydroxycarbonyl (Boc) and p-benzyloxymethyl (Bom) groups were used. Alanine (Ala) residue was introduced at the C-terminus of the dendrimer. *N*ε, *N*α-di-(tert-butylhydroxycarbonyl)lysine was introduced into the branching points and, subsequently, double the amount of amino acid derivatives were added. 4-*N*, *N*-dimethylaminopyridine (DMAP) was added to the reaction mixture as a catalyst for complete conversion on the last stage of the dendrimer growth. The effectiveness of coupling was checked by performing the Kaiser test. Then the resin was rinsed with DMF and dichloromethane (DCM). At the final stage of the synthesis the target dendrimer was cleaved from the polymeric carrier with complete deprotection of the TFMSA (1 mL)/TFA (10 mL) system in the presence of scavengers. After filtration the Lys-2His dendrimer was precipitated in ethyl ether (300 ml), centrifuged, and dried under vacuum.

Preliminary purification of the crude dendrimer was performed by gel-filtration on Sephadex G-50 column (2.5 × 50 cm) using 10% acetic acid as eluent. The corresponding fraction was dialyzed against distilled water in a dialysis membrane bag (MWCO 1000) for one day. After two days of freeze drying, Lys-2His dendrimer was collected. Purification degree of the product was analyzed by RP-HPLC in the water–acetonitrile–0.1% trifluoroacetic acid system using linear ascending acetonitrile gradient. The dendrimer fraction had a purity of 95%.

Thus, the general protocol includes the following stages: (1) deprotection, 70% TFA/CH_2Cl_2 (10 ml), 15 min; (2) washing, CH_2Cl_2 (10 ml × 2), DMF (10 ml

× 2); (3) deprotonation, 10% Et_3N/DMF (10 ml × 2), (4) washing, DMF, (10 ml × 2); (5) coupling, 1.5 mM Boc-amino acid, 15 mM DIC, 1.5 mM HOBt/DMF (10 ml); (6) washing, DMF (10 ml × 2), CH_2Cl_2 (10 ml × 2); (7) the Kaiser test. In the case of incomplete coupling (positive ninhydrin reaction) the protocol is repeated from stage (3).

The principal scheme of the synthesis of Lys-2His dendrimer from the core to the first generation is presented in Fig. S1.

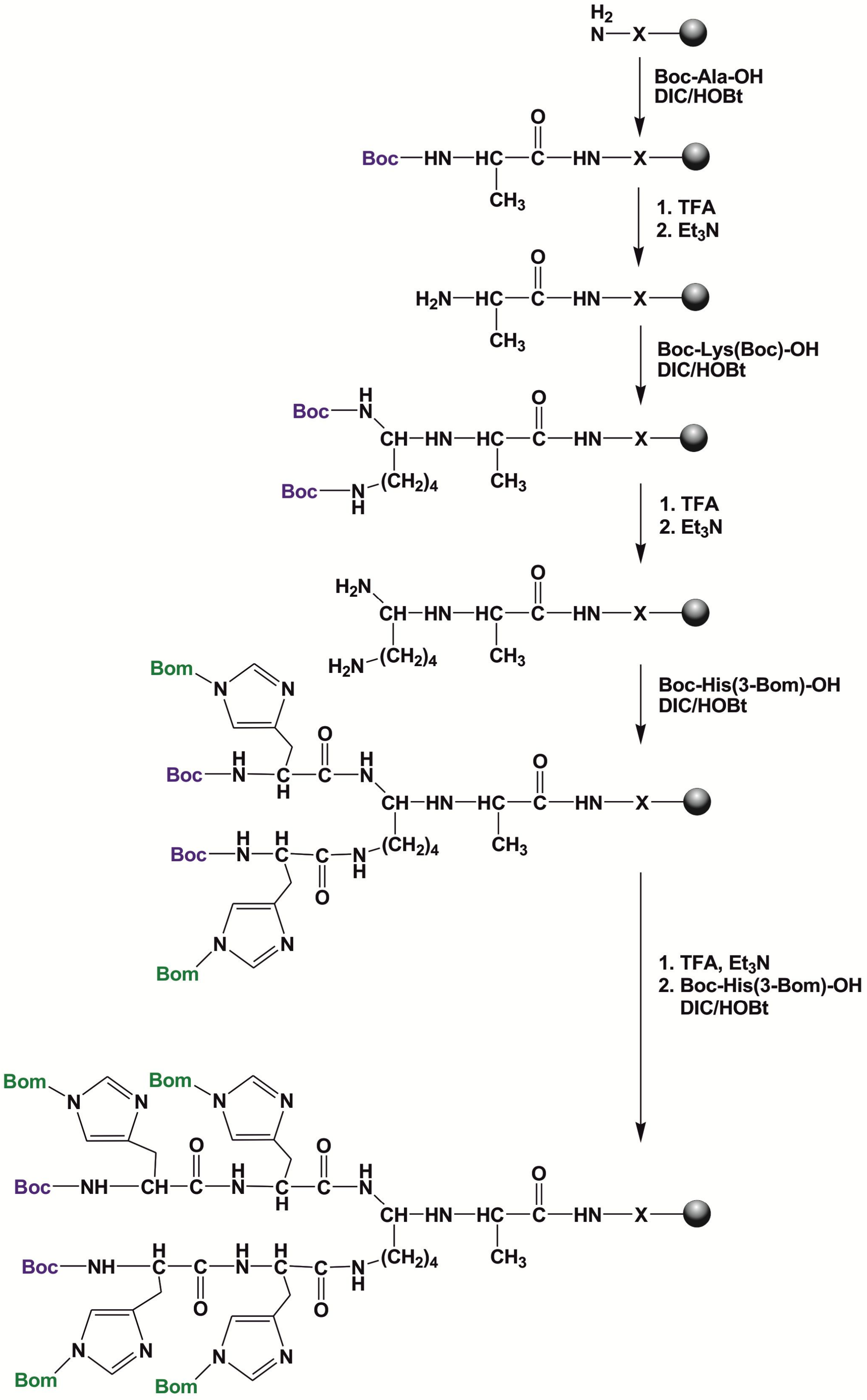

Figure S1. *The principal scheme of the synthesis of Lys-2His dendrimer from the core to the first generation.*

^1H and ^{13}C NMR spectroscopy analysis

We present the two-dimensional ^1H-^{13}C HSQC (Fig. S2) and HMBC (Fig. S3 and S4) NMR spectra of Lys-2His dendrimer to analyze its structure in detail.

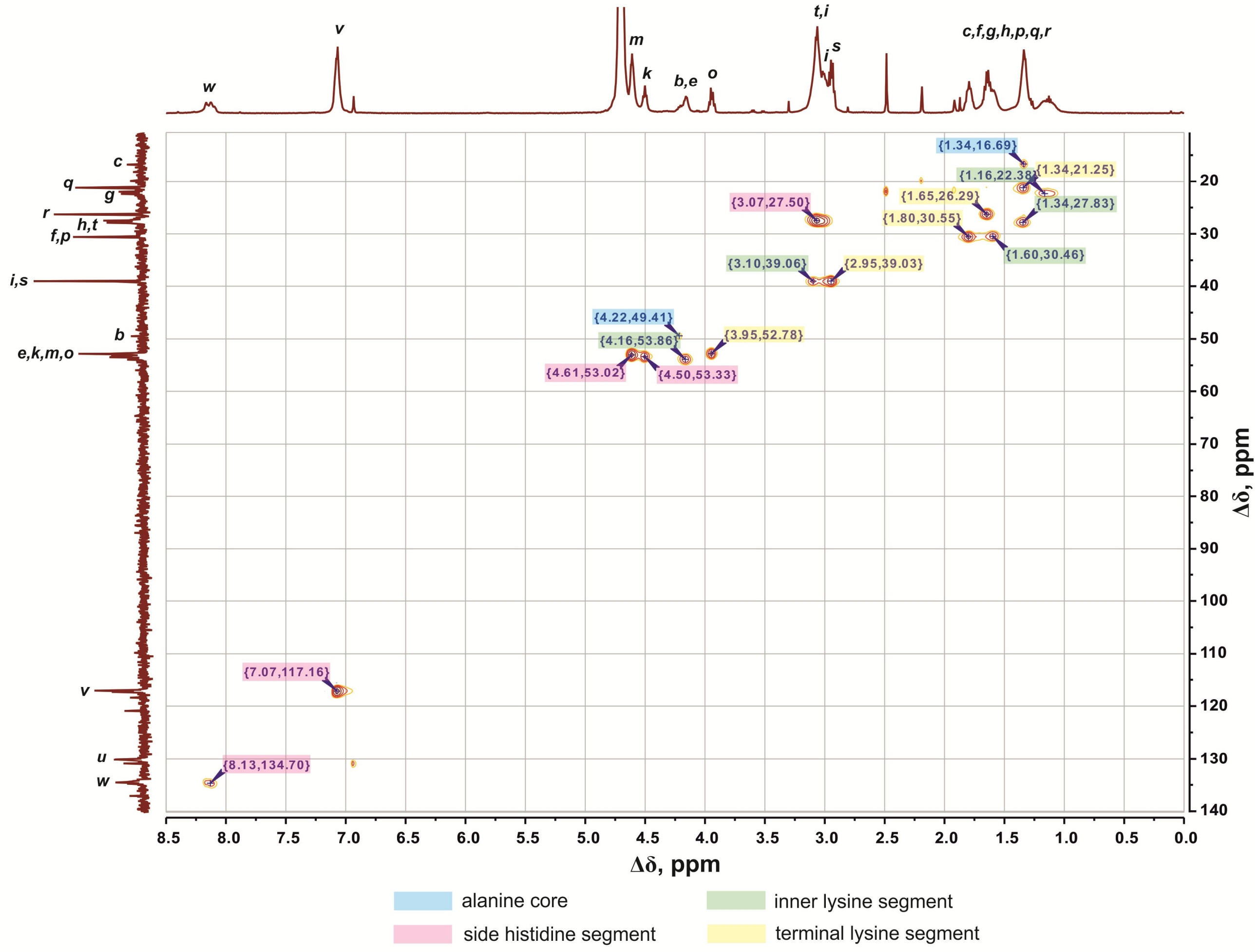

Figure S2. *^1H-^{13}C HSQC spectrum of Lys-2His dendrimer in D_2O at 25 °C. The letter symbols correspond to the designations of the groups in Fig. 2.*

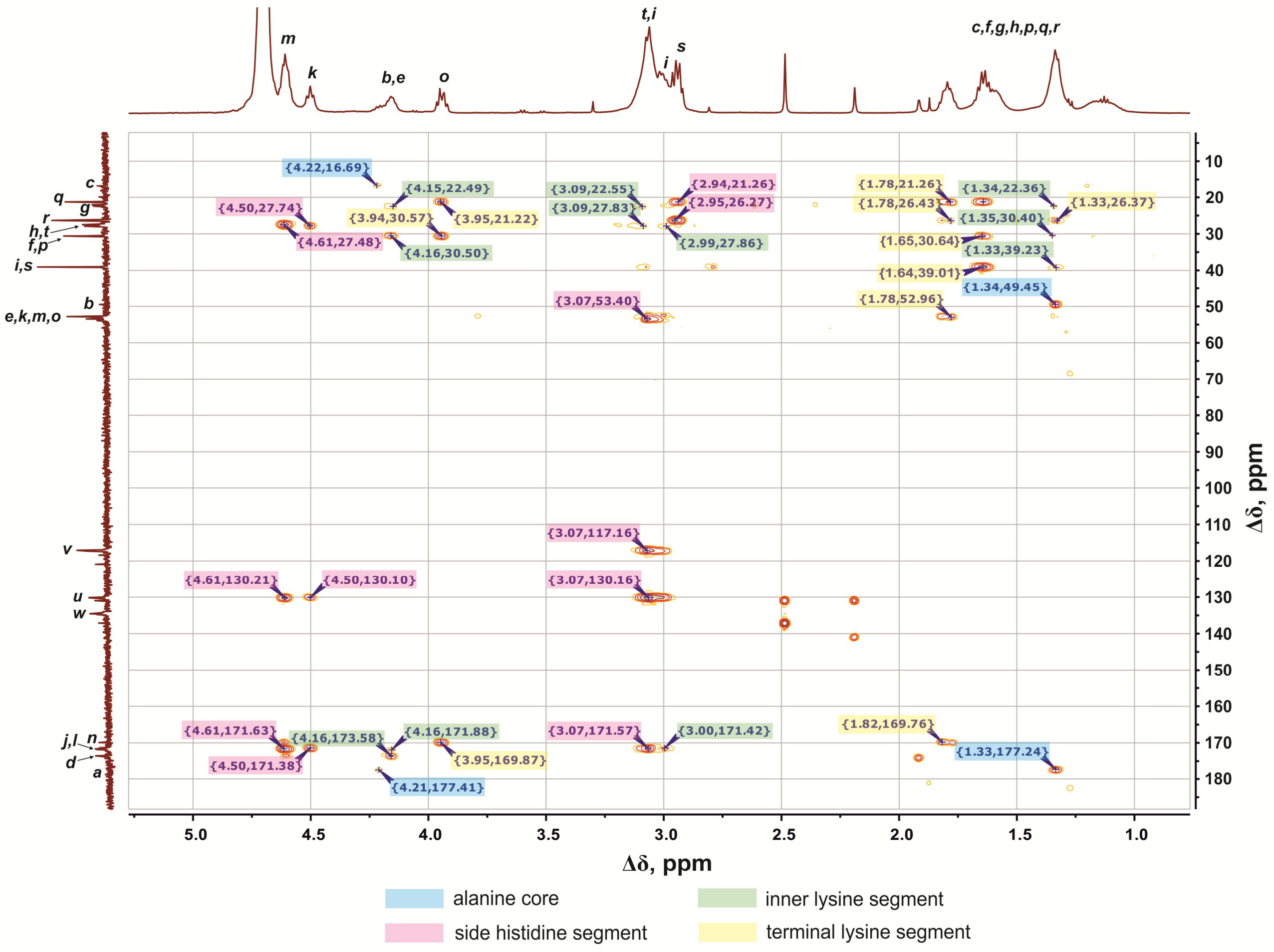

Figure S3. *^1H-^{13}C HMBC spectrum of Lys-2His dendrimer in D_2O at 25 °C at the range from 5.0 ppm to 1.0 ppm. The letter symbols correspond to the designations of the groups in Fig. 1.*

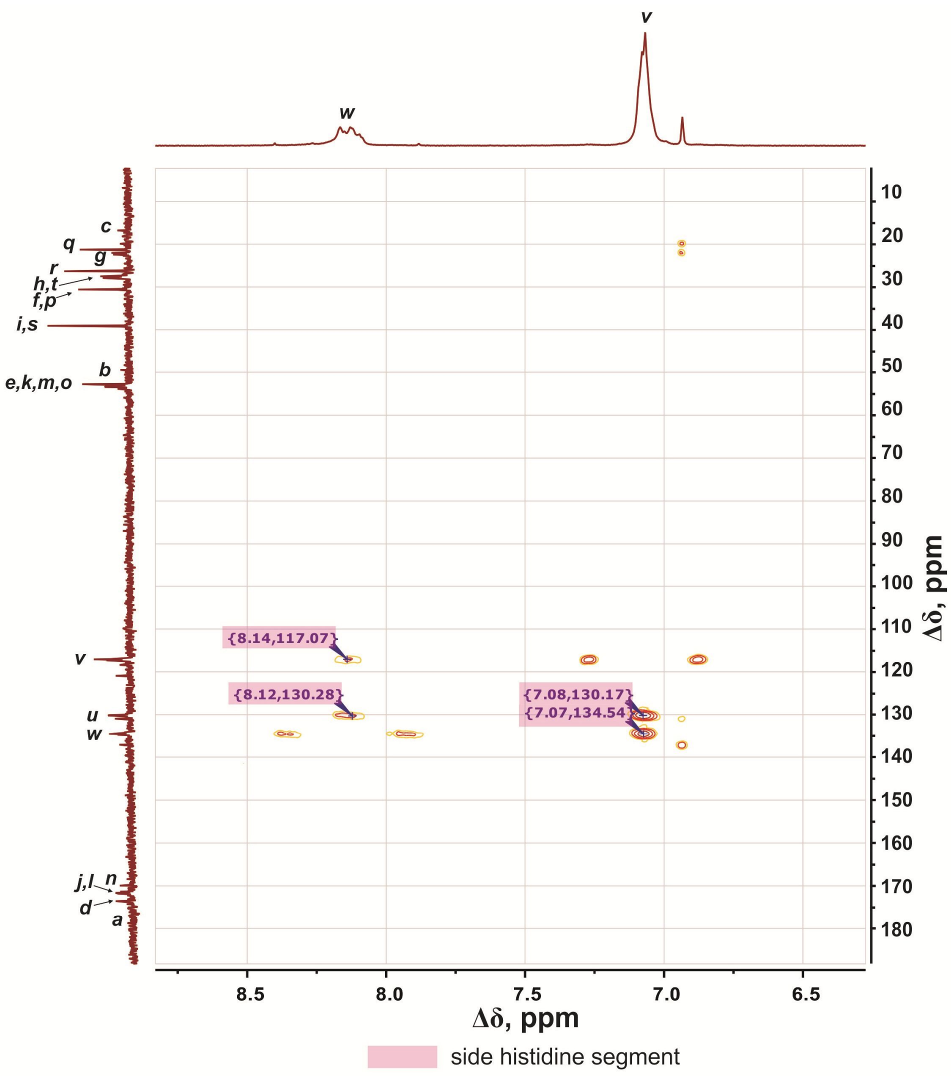

Figure S4. *^{1}H-^{13}C HMBC spectrum of Lys-2His dendrimer in D_2O at 25* °C *at the range from 8.5 ppm to 6.5 ppm. The letter symbols correspond to the designations of the groups in Fig. 1.*
